# Obstetricians’ views on extending the 12-week abortion limit in Belgium: A qualitative study

**DOI:** 10.1371/journal.pone.0325434

**Published:** 2025-06-18

**Authors:** Fien De Meyer, Kim Beernaert, Sarah Van de Velde, Sigrid Sterckx, Kristof Van Assche

**Affiliations:** 1 Research Group Personal Rights and Property Rights, Faculty of Law, University of Antwerp, Antwerp, Belgium; 2 End-of-Life Care Research Group, Vrije Universiteit Brussel & Ghent University, Ghent, Belgium; 3 Centre for Population, Family and Health, Department of Sociology, Faculty of Social Sciences, University of Antwerp, Antwerp, Belgium; 4 Bioethics Institute Ghent, Department of Philosophy and Moral Sciences, Ghent University, Ghent, Belgium; 5 Research Group Personal Rights and Property Rights, Faculty of Law, University of Antwerp, Antwerp, Belgium; World Health Organization, SWITZERLAND

## Abstract

**Aim:**

This study explores the views of obstetricians on a potential extension of the 12-week limit on elective abortion in Belgium. It aims to investigate perspectives from a distinct group of providers who already have experience with later abortion, albeit provided on medical grounds. These insights could help identify and navigate perceived challenges and opportunities of extending the abortion time limit.

**Methods:**

We conducted semi-structured qualitative interviews with 23 hospital obstetricians who are involved in abortion on medical grounds in Flanders, Belgium. We coded and thematically analyzed interview transcripts with NVivo software.

**Results:**

We discerned three major themes in obstetricians’ views: 1) Concerns about later abortion techniques; 2) Psychosocial hardships justifying later abortion; and 3) Fetal viability and feticide considerations. Minor themes are: 4) Limited impact on abortion for fetal anomaly; 5) Abortion travel and associated harms; and 6) Sex-selective abortion.

**Conclusion:**

Flemish hospital obstetricians highlight both moral and technical challenges associated with extending the abortion time limit, primarily relating to surgical abortion methods and the organization of care. This study finds that, if an extended limit were to be adopted, additional policies and safeguards would facilitate implementation and foster interest among obstetricians in providing services. These include education and technical training of providers in later abortion care, centralization of provision, appropriate facilities, prepared teams, value workshops, and adequate state financing.

## Introduction

Timing is crucial for abortion access. Laws and policies often restrict abortion through gestational age limits, commonly prohibiting access beyond the first trimester [1–5]. In Belgium, elective abortion – abortion upon request, i.e., where the person is not required to provide any justification under the law – is permitted up to 12 weeks after conception [6]. Beyond 12 weeks, abortion is only permitted for severe medical conditions, specifically when there is “a particularly serious and incurable fetal condition” or “a serious threat to the woman’s health”. Each year, between 400 and 500 Belgian women travel to the Netherlands for abortions up to 22 weeks. In 2019, a legislative proposal to extend Belgium’s gestational age limit to 18 garnered support from several political parties, yet failed to pass into law [7,8].

In the Belgian region of Flanders, abortion clinics support extending the limit, but obstetricians (who are primarily involved in terminating pregnancies for severe health conditions) are more critical [9,10]. No prior research has studied abortion providers’ views and attitudes towards a potential abortion time limit extension in Belgium. Internationally, health professionals’ perspectives on (providing) abortion after the first trimester are underexplored [11–14]. Studies from South Africa, where elective abortion is also legal up to 12 weeks, suggest that while abortion providers recognize the importance of later-stage abortion access, they feel uncomfortable about providing it [12,15,16]. In particular, there is little research on obstetricians’ views on legalizing elective abortion beyond 12 weeks. In many countries, obstetricians routinely manage abortions after the first trimester, albeit exclusively for medical reasons. Their insights could highlight practical challenges and inform service organization if the law changes.

This study explores Belgian hospital obstetricians’ views on legalizing elective abortion beyond the first trimester of pregnancy (after 12 weeks). We aim to understand potential concerns and explore how an extended limit might affect the obstetric profession. These findings could guide policymakers and healthcare providers on this issue.

## Methods

### Study design

We conducted semi-structured interviews with hospital obstetricians in Flanders, Belgium. As part of a broader study, our interviews explored obstetricians’ decision-making processes regarding termination of pregnancy for medical reasons and their perspectives on the abortion law, including the time limit for elective abortion. We carried out 23 interviews between October 1, 2020 and April 30, 2021.

### Population and setting

Our study included obstetricians working in hospitals in Flanders or in the bilingual region of Brussels in Belgium. These hospital obstetricians are primarily involved in termination of pregnancy beyond the first trimester of pregnancy on medical grounds. Most elective abortions are currently performed in specialized abortion clinics. However, in rare cases (e.g., elevated patient risk, anesthesia requests, or early fetal anomaly detection), obstetricians operating in hospitals handle these requests. After 12 weeks, abortions (on medical grounds) are exclusively performed in hospitals under the supervision of obstetricians. The common method to terminate these second and third trimester pregnancies is through medication (known as ‘medical abortion’). Medical abortion concerns induction of labour using a combination of medicines, taken either orally or vaginally. There is currently no established practice in Belgium of using surgical abortion (known as dilation and evacuation or ‘D&E’) in second trimester pregnancies.

### Sampling and recruitment

With the use of the research team’s personal network and through a targeted internet search, we purposively contacted a total of 40 hospital obstetricians through email. To capture a wide variety of institutional practices and views, we selected participants from both hospitals with and without a neonatal intensive care unit, i.e., ‘academic’ hospitals vs. ‘regional’ hospitals. We focused on recruiting obstetricians who had previously been involved in decision-making on termination of pregnancy after 12 weeks on medical grounds. For obstetricians operating in regional hospitals, we used two additional recruitment methods. First, we requested heads of department to put us into contact with potential participants through email. Second, we used the “snowballing sampling method” at the end of interviews to recruit additional participants.

### Data collection

The multidisciplinary research team developed a semi-structured topic guide, which was consistently used across all interviews. An external obstetrician gave feedback on the topic guide. Each interview was conducted by a duo comprising the first author (FDM) and one of the co-authors – a psychologist, ethicist, or sociologist (KB, SS, or SVDV). Participants were given the option to take part in either in-person or digital interviews. 21 out of 23 interviews took place online due to COVID-19 safety restrictions. For these interviews, we used Microsoft Teams, a secure video conferencing platform supported by the team’s academic institutions. By using this institutional platform along with a linked, password-protected storage drive, we ensured that sensitive data were securely stored. Interviews were conducted in private settings to maintain confidentiality, although some participants experienced inevitable and occasional interruptions from colleagues. We recorded all interviews and used a professional transcription service, bound by a confidentiality agreement, to transcribe them. At the start of each interview session, participants also completed a brief questionnaire on their sociodemographic and professional characteristics (details provided in Table 1, *infra*).

**Table 1:** Characteristics of obstetricians who participated in the study.

	N
Age	
30-39	8
40-49	5
≥ 50	10
Gender	
Male	8
Female	15
Years of professional experience	
< 5	1
5-10	6
11-19	6
≥ 20	11
Frequency of involvement in TOP counselling^a^	
on a weekly basis	13
on a monthly basis	5
less than on a monthly basis	5
Involved in feticide^b^	
No	15
Yes	8
Hospital type	
Non-academic	12
Academic	11

^a^Frequency of obstetricians’ current participation in decision-making and counselling for pregnancy terminations after 12 weeks, permitted only on medical grounds.

^b^Whether the obstetrician performed feticide – a specialized procedure in later-stage abortions aimed at ensuring fetal demise by inducing cardiac arrest. Those who did not previously perform feticide had all managed medical abortions after 12 weeks, conducted for fetal or maternal health indications, in their role as treating obstetricians.

The team initiated coding after the first five interviews to facilitate familiarization with the data and to refine the interview topic guide where necessary. Thematic data saturation was assessed inductively through regular team discussions during the data analysis phase (see *infra*). Based on input from the principal researcher, who attended all interviews, the team concluded that no substantially new codes or themes were emerging in the later stages of data collection, indicating that thematic saturation had been reached.

The first part of the interview focused on obstetricians’ decision-making processes regarding medically indicated abortions [17]. The second part explored their experiences with and views on abortion law, with particular attention to the current 12-week limit for elective abortions and the proposed extension of this limit. Through open-ended questioning, we investigated how obstetricians view the 12-week elective abortion limit and its proposed extension. While this article primarily presents participants’ responses drawn from the second section of the interview, it also includes relevant insights from the first section. This is because obstetricians often raised issues related to the 12-week limit and non-medically indicated abortions − typically referred to by them as ‘social abortions’ – in their discussions of abortion requested on medical grounds. To ensure consistent terminology, this paper uses the term *elective abortion* to refer to abortions requested or permitted before the 12-week legal limit for various reasons − including social, economic, relational, mental, or other − as opposed to *abortion on medical grounds*, referencing abortion requested or permitted because of a serious fetal or maternal health condition.

### Data analysis

Guided by thematic analysis [18] and led by the principal researcher, the research team collectively coded and analyzed the transcripts. The research team used NVivo software to conduct the analysis. One researcher also manually coded the transcripts to ensure reliability and minimize potential bias from a single coding method. The principal researcher initiated coding after the first five interviews to generate initial codes. Through regular theme discussions, themes were identified, reviewed, refined, and defined. The principal researcher coded all transcripts to maintain a comprehensive overview of emerging themes, while four additional team members each coded approximately six transcripts. This process ensured that every transcript was at least double-coded. The principal researcher compared coding results across team members, resolving ambiguities within the larger team. All quotes presented in this paper were translated from Dutch to English, with careful attention to preserving their literal phrasing and original meaning.

### Ethical considerations

Prior to the interview written informed consent was obtained from all the participants in the study. We were granted ethical clearance from the ethics committees of University Hospital Antwerp (no. 20/31/407) and University Hospital Ghent (no. BC-08291). Conducting private interviews provided participants the opportunity to express their views openly, with confidentiality further ensured through the pseudonymization of transcripts.

### Reflexivity

In conducting this study, we recognize that our diverse backgrounds and perspectives inevitably shape aspects of the research process, including data interpretation. For example, the first author, a woman, reproductive rights supporter, and legal researcher, approached this work with a genuine interest in advancing legal and policy discussions around abortion, particularly in examining potential extensions of the abortion time limit. This perspective may have influenced the focus on addressing challenges identified by participants and considering actionable pathways for policy enhancement. Furthermore, although we used a standardized topic guide, the interviewers’ diverse academic backgrounds sometimes led to unique emphases in interview prompts. For instance, some of the team’s researchers engaged participants more on moral and philosophical questions, others on legal mechanisms for change, and still others on the sociological factors behind later presentations for abortion. To maintain consistency, the principal researcher attended all interviews, observing and addressing any varying emphasis to align with the study’s core objectives. We are aware that our positionalities – individual perspectives and professional orientations – may have influenced our understanding of the obstetricians’ perspectives on elective abortion timing. However, we believe that the team’s multidisciplinary makeup, regular feedback sessions, and the rotation of interview pairings helped guard against unintentional bias. These strategies promoted a balanced approach, enabling us to interrogate the data critically while respecting the diverse viewpoints shared by the respondents.

## Results

We conducted interviews with 23 hospital obstetricians working in Flanders, Belgium, between October 1, 2020, and April 30, 2021. Each interview lasted approximately 90 minutes, with about one-third of the time dedicated to discussing time limits for elective abortion and the potential extension of those limits. Personal and professional characteristics of the participating obstetricians are summarized in Table 1.

Overall, we found that obstetricians refrained from discussing their personal views regarding the elective abortion time limit and the need to allow it after the first 12 weeks of pregnancy. Our participants submitted that elective abortions were not their hospitals’ core activity and frequently distanced themselves from this type of abortion provision. The small number of participants who did express personal views shared opposing views, either supporting or disapproving of elective abortion after 12 weeks of pregnancy. Moreover, as a general observation, we found that the obstetricians did not base their considerations regarding a potential extension of the 12-week abortion limit on ideological, philosophical, or rights-based arguments. For example, there was minimal reference to concepts such as bodily autonomy or gender equality, and little engagement with philosophical or theological perspectives on the presumed moral status of the fetus. Rather than considering *if* and *why* a time limit extension should be considered, our respondents focused on medical, technical, and logistical arguments, frequently paired with ethical concerns about performing specific abortion techniques after the first trimester of pregnancy.

We identified six key themes in our analysis of obstetricians’ views. We differentiate between major and minor themes based on several factors: the frequency with which themes recurred across interviews, the extent to which obstetricians spontaneously introduced these themes, and the importance that obstetricians appeared to assign to them. Major themes are: 1) Concerns about later abortion techniques; 2) Psychosocial hardships justifying later abortion; and 3) Fetal viability and feticide considerations. Minor themes are: 4) Limited impact on abortion for fetal anomaly; 5) Abortion travel and associated harms; and 6) Sex-selective abortion.

### Major themes

#### Concerns about later abortion techniques.

Nearly all participants explicitly expressed concerns over resorting to surgical abortion, also known as dilation and evacuation or ‘D&E’, due to its perceived technical and moral difficulty. Obstetricians found the medical abortion method superior to surgical D&E, at a minimum in the context of severe medical conditions. Although participants implicitly recognized that surgical abortion would be a more appropriate method to use in the context of elective abortion, they stressed the providers’ difficulties with the procedure.

*“Sorry, but those curettages, how they do that at 14 weeks... We as obstetricians, after 12 weeks, have to have a lot of courage to do that. (…) That’s just ethically difficult, come on, you need a piece of head, arm, leg, (…) Have you ever seen a fetus at 15, 16 weeks? That’s a small person. (…) I find that unacceptable.” – Participant 18*

*“If you do it with medication and just induction, (…) it isn’t that horrible, is it? I mean, people are confronted with the fact that it takes time, that you have to go through it. That you have to experience it completely. (…) If you do it through a mechanical abortion, then it is really rough butchery, I mean, nobody likes to do that, I think” – Participant 9*

Participants alluded to the technical complexity of surgical abortion after 12 weeks and the lack of expertise among Belgian obstetricians in performing these techniques. Participants sometimes drew comparisons with the expertise in the Netherlands, where elective abortions have long been legal until viability and are commonly performed using D&E.

“*I’m used to a lot anyway, but I also don’t like to perform an abortion at fourteen weeks. Those are difficult curettages, that is actually not easy.”* – Participant 15

*“(…) in the Netherlands (…) dilations and evacuations are commonplace. That is a well-known method, also performed by people who have a lot of experience. I challenge you to find someone in Belgium who has performed more than ten such procedures in the last two years.” – Participant 10*

While less prominent in the discourse than the moral and technical implications of surgical abortion methods in later pregnancy, participants also highlighted the potential negative impacts on the health of patients undergoing later abortion procedures. Health risks mentioned include excessive bleeding, compromised future fertility, and psychological burdens. Most of these risks were mentioned in reference to D&E, although some suggested that the long duration and ‘full experience’ of a medical abortion procedure could potentially compromise the health of the abortion-seeker.

*“It is also not without risk for the mother. You also have to be a bit consistent in that every now and then you can have a complication that is a bit more serious for the future of that woman. (…) We do curettages here up to 16 weeks, let’s just say I’m the only one who dares. This can bleed very severely, and so on. And quietly I teach it to others too, the tricks to go through it safely, but after 16 weeks I think it’s really very risky and I prefer to do it medically (…).” – Participant 4*

*“I think we do have some reservations about that [time limit extension] or are a bit afraid. We already see that (…) some people sometimes come back after abortion with complications simply from the procedure, and we also see how much time we spend on [medical] terminations of up to 18 weeks or up to 20 weeks or wherever the limit will go (…). There can certainly be problems with that, both physically, obstetrically, but also on an emotional and psychological level.” – Participant 8*

Given these perceived challenges, most obstetricians’ support for a time limit extension beyond 12 weeks appeared to be either absent or conditional upon safeguards for an adequate organization of abortion care. The safeguards mentioned included the centralization of second trimester abortion provision, training and education regarding use of surgical methods, experienced and supportive teams, and adequate financial support from the government.

*“When we start to terminate a pregnancy up to and including 18 weeks, we need to be given the means to do that. We’re very limited in terms of staffing, in terms of infrastructure, so we have to get the resources. If we get that, we can leave that door open.” – Participant 22*

*“I’m in favor of it being extended, but I’m also in favor of adequate training and proper centralization, so that it happens in a hospital where the team knows it well, is prepared for it, both physically* – *knowing procedurally what you have to do and what you can do if this or that doesn’t work* –*, and also psychologically. (…) And that we have one central location per province, preferably connected to a hospital. (…) If all that is in order, I’m all in favor and I even want to join in.” – Participant 12*

#### Psychosocial hardships justifying later abortion.

Obstetricians were generally more supportive of legalizing second trimester abortion in cases where a psychosocial component or justification is present. Examples of psychosocial justifications mentioned include pregnancy from rape, teen pregnancy, severely compromised socio-economic background, difficult divorce, background of serious psychiatric issues and drug addiction, and combinations of these circumstances. Some participants presented psychosocial hardships as inherent to most abortion requests at later gestational ages; others viewed them as exceptional circumstances justifying these requests.

*“And I can guarantee you, that [a request for later abortion] is not because they simply don’t want a child; they have very serious social problems.” – Participant 17*

*“I think for such late abortions that you have to develop the same procedure as for therapeutic abortion. (…) Of course, there must be an important psychosocial reason, a psychosocial disability, let’s say.” – Participant 7*

Due to the assumed medical component in psychosocial abortion requests after 12 weeks, some obstetricians proposed handling these cases similarly to abortion requests on medical grounds. This approach would eliminate the need for a blanket extension of the time limit for *all* elective abortions. Despite support for assisting individuals facing severe psychosocial issues, the obstetricians highlighted the personal and legal difficulties in defining and assessing the psychosocial issues that might justify later abortion.

*“I think if you really have those exceptional situations, we could make a transition rule in which we say: all those who are in such a dramatic situation and who present (…) after fourteen weeks; let them appear before a committee in Flanders to decide on this. (…) But to suddenly extend the law to eighteen or twenty weeks…” – Participant 10*

*“I do think it should be looked at carefully (…) how pronounced and how real that [later abortion request] is. I can’t judge that either, I’m really not good at that.” – Participant 18*

A smaller number of obstetricians confirmed that abortions in a context of severe psychosocial difficulties of the pregnant individual had, in the past, already been offered in their hospital after the first trimester of pregnancy as an option of last resort.

*“We had, I think not too long ago, a fourteen-year-old girl, who had an unplanned pregnancy and a significant psychiatric burden, [including] suicidality. I mean, really, really, really very sad, and she was also fifteen weeks I think.” – Participant 8*

Despite these testimonies, obstetricians were divided on whether such situations would genuinely be qualified as a legal abortion due to ‘a serious threat to the pregnant woman’s health’ – the ground in the current Belgian abortion law that permits abortion after 12 weeks.

*“Someone who has severe cardiomyopathy whom we know will not survive the pregnancy or has a one-in-two chance (...). That is what it [the law] covers. However, it does not cover psychiatry; it does not cover (...) rape; it does not.” – Participant 18*

#### Fetal viability and feticide considerations.

Approaching viability and feticide limits represented an ethical counterindication for the obstetricians in the consideration of legalizing elective abortion beyond the first trimester.

*“If you’re going to shift it to time limits that are going to approach viability, then you’re going to have more colleagues who have an ethical problem to perform the procedure. Rightly so, I think.” – Participant 21*

Participants expressed moral distress about terminating a healthy pregnancy near or beyond the viability threshold. This distress often related to the perceived need to perform feticide, which avoids signs of life following a medically induced expulsion of a premature fetus. Additionally, though less regularly, some participants raised concerns about the fetus’s pain capacity in later-stage terminations.

*“I’ve done a few under ultrasound guidance and, for example, for me, a feticide has to be done before the ‘morcellation’. There are more and more arguments indicating that pain awareness in the fetus can be developed earlier, it is not yet 100% sure from when. Are we talking about 22 weeks, are we talking about 18 weeks…?” – Participant 22*

In line with the above statements, participants who were open to extending the 12-week limit preferred limits at or shortly before fetal viability and/or pain thresholds as alternative limits in the hypothesis of extension.

*“You could take the limit of viability that is accepted at that moment, and you could possibly say, you know what, let’s subtract 2 weeks, because in America there will always be a fetus of 22 weeks that they will be able to pull through. So, no problem, you can easily say 20 weeks. Twenty weeks is exactly half the pregnancy, there are no known cases where a child can survive at twenty weeks.” – Participant 19*

*“I think that’s kind of the border for feticide (…) you can’t just push those children through a labor (…) or force them into life. Considering also that the fetus can feel pain from 22 weeks. 22, I think that’s kind of the limit for me, that’s where I draw the line.” – Participant 18*

### Minor themes

#### Limited impact on abortion for fetal anomaly.

Obstetricians generally distanced themselves from elective abortion and the time limit debate, focusing on medically indicated abortion instead, to which no maximum time limit applies. Because of their limited involvement in elective abortion, most participants did not perceive the abortion time limit as relevant or limiting to them. Similarly, they rarely reflected on potential implications of a time limit extension for their own core activity, i.e., providing obstetric care and termination of pregnancy in case of fetal anomaly.

Nevertheless, sporadic statements of obstetricians countered the general perception that an extended elective abortion limit would not affect in-hospital termination of pregnancy services and the obstetric profession. Some participants discerned a role for hospitals in offering later abortions upon request after the anticipated legal reform due to the safety, infrastructure, and quality standards that hospitals meet, suggesting a more pragmatic link between both types of abortion care. Some others mentioned a potential impact on the obstetric profession in the context of care trajectories surrounding ‘milder’ fetal anomalies. For instance, some considered an extension of the time limit useful to avoid discussions on whether a fetal anomaly was ‘sufficiently severe’ to allow abortion on medical grounds, or to expedite termination of pregnancy for clear-cut conditions.

*“What may be the case is when you have a deviating NIPT [non-invasive prenatal test] or you see something on your first trimester ultrasound and you can’t do a puncture until 16 weeks, and these result in something genetically vague, that we would no longer terminate (…). But those people are then also in the cold for a social abortion. Maybe that is a situation in which you could discuss deviating from that 14-week limit a little bit so that those puncture results can still be taken into account by the parents.” – Participant 13*

*“Maybe it’s good to keep it before twenty weeks. Because at twenty weeks, there’s another ultrasound, and sometimes, even the tiniest things – things that aren’t bad at all, just a little bit different – can really cause people to panic. (…) And then I wouldn’t want someone, out of misunderstanding and being too blunt, to say, ‘No, we’re not going through with this.’” – Participant 12*

Participants generally did not fear an increase in abortion requests for ‘mild’ fetal health problems in the hypothesis of a time limit extension for elective abortion. Those who reflected on this argument expressed strong faith in the current medical-ethical decision-making at the level of hospitals, regardless of a possible change of the law.

*“I think we would still get the same questions for the same anomalies, because everyone judges for themselves what is desirable and what is not and what is acceptable and what is not. (…) I just think that if you have a good system, where there is registration, then you can’t just say, we’re going to terminate (…) for a cleft lip. You should always know that you are in a system, aren’t you? You are in a structure.” – Participant 18*

#### Abortion travel and associated harms.

Only a small number of participants reflected on abortion travel and its associated harms for those seeking abortions. Among these participants, opinions varied regarding the significance of abortion travel in the debate on extending the elective abortion limit.

For instance, a few participants referred to out-of-country referral and consequent abortion travel after the first trimester as traumatizing and not patient-friendly.

*“(…) there are approximately 500 women each year who go through the trauma of having to travel to the Netherlands with their ‘child’ at 16-17-18 weeks, to have an abortion on social grounds.”* – Participant 7

Some others did not mention or value abortion travel and associated harms as compelling arguments in the discussion. One participant, in particular, appeared to regard abortion travel and its associated hardships as justified consequences of seeking an abortion at a later stage.

*“If you can send money to another country, should Belgium suddenly become a fiscal paradise too? Let at least a part of them go [abroad], let them have a harder time, let them think about it. Why do we always have to do everything?”* – Participant 9

#### Sex-selective abortion.

Most participants did not spontaneously touch upon a potential impact of an extended abortion limit on sex-selective abortion [19]. However, as it is a common argument in the political debate on abortion time limits, they were invited to reflect upon the topic’s relevance through prompts. A limited number of obstetricians believed that sex-selective abortion could become a greater issue under a time limit extension, at least in theory. These participants stressed the rarity of such requests.

*“You will unfortunately have a small fraction [of people] who will abuse it for the sake of sex selection. I think that is, for the people who write the law, possibly a problem, if you know that exists.” – Participant 19*

“*Disappointed? Yes, I see them every now and then. But pregnancy terminations? (…) I do not believe that that will be the great bulk of pregnancy terminations [if the time limit were to be extended], that seems very unlikely to me, that is very far-fetched.” – Participant 10*

Although considered rare, some obstetricians noted that abortion requests for reasons of sex selection already occur under the current law and are therefore impossible to eliminate entirely. This observation diminished the relevance of sex-selective abortion as an argument against extending the time limit.

*“In principle you can already do it now. Because I know some women lie about their pregnancy age and then have a NIPT done at 11 weeks and then have the result at 12 weeks. (…) It happens and I’ve seen women do that. I’m convinced that will not increase.” – Participant 2*

## Discussion

This study provides insight into the perspectives of Belgian hospital obstetricians in Flanders, Belgium, regarding extending the current 12-week legal limit for elective abortion. Rather than focusing on their personal views about whether or why a time limit extension should be considered, our respondents emphasized medical, technical, and logistical considerations, often accompanied by moral concerns about performing specific abortion techniques beyond the first trimester. In this discussion, we interpret these findings in the context of relevant literature and the Belgian abortion law. We focus on three key discussion points, revolving around 1) moral and clinical concerns regarding later abortion techniques; 2) the social and structural realities behind later abortion; and 3) the classification of abortion and abortion refusal.

### Moral and clinical concerns regarding later abortion techniques

The first major theme identified in this study involves obstetricians’ moral and clinical concerns regarding the use of surgical techniques beyond the first trimester. Dilation and evacuation is the main method to terminate second trimester pregnancies in countries that allow these terminations on social grounds or on request, including in the Netherlands, the United Kingdom, and the United States. During a D&E procedure, the cervix is medically dilated over a period of 24–48 hours, and fetal tissue is later removed from the uterus using forceps. Although the literature is limited, studies from other countries have reported similarly reserved views among unfamiliarized healthcare providers and maternal-fetal medicine specialists on providing surgical abortion after the first trimester [15,16,20,21]. Our participants’ moral and clinical reservations with D&E were often, but not necessarily, associated with negative views towards extending the time limit for elective abortion. In fact, moral ambivalence towards D&E and/or personal involvement in abortion provision sometimes went hand in hand with principled support for second trimester service delivery. Such “mixed” attitudes and testimonies from health professionals have also been identified in the literature [15,22].

Furthermore, we observed that concerns about health risks and potential complications associated with later abortion techniques, particularly surgical techniques, complicated support for extending the gestational time limit. Studies confirm that second trimester abortion carries increased health risks compared to first trimester abortion. However, these studies simultaneously consider the absolute risks low [23–25]. More specifically, an increasing body of research from settings where D&E is commonly practiced indicates that D&E has overall lower complication rates, is more effective, quicker, preferred by women and associated with better emotional coping than medical abortion in the second trimester [24,26–32]. Researchers have emphasized the complex interplay of factors that influence the suitability of a particular abortion method in a given situation, highlighting the critical importance of providing comprehensive information to abortion-seekers and empowering them to make informed choices [27,33]. Notably, these insights into safety and suitability of abortion methods were not explicitly addressed or considered by the obstetricians during interviews. One possible explanation is the shortage of trained providers and suitable facilities in Belgium – both essential prerequisites for ensuring the safety of the procedure and minimizing complications associated with D&E. Research confirms that safe D&E requires skilled personnel, specialized instruments, and a sufficient caseload to maintain proficiency [34]. Apart from technical training, another study recommends workshops aimed at clarifying values and transforming attitudes to support empathetic abortion care and to help identify which providers are willing to pursue specialized clinical training in second-trimester elective abortion [35]. These workshops aim to address access barriers caused by misinformation and stigma by guiding healthcare providers through a process of self-reflection, helping them distinguish their personal values from the needs and rights of individuals seeking abortion care. The safeguards identified in this paper seem crucial for effective implementation if the proposed legal reform would be adopted in Belgium, ensuring a trained, sufficiently large, and willing workforce, while fostering interest among obstetricians in providing such services.

Beyond the challenges associated with the use of dilation and evacuation, other key moral and clinical considerations in the time limit debate revolved around fetal viability, feticide, and to a lesser extent, fetal pain. Obstetricians opposed extending the elective abortion limit near or beyond these thresholds. Regarding viability, studies argue that it should not determine the justifiability of abortion, emphasizing that viability is a medical concept for guiding neonatal care, not for regulating pregnancy termination [36,37]. These further highlight that prioritizing fetal viability risks overlooking the rights and circumstances of pregnant individuals [37]. In our study, obstetricians engaged with viability not only as a biological or clinical concept but also as a threshold reflecting some form of moral status of the fetus, consistent with a recent study identifying viability as a significant moral value in obstetricians’ decision-making in abortion care [38]. Policymakers considering a time limit extension should be aware of, and carefully weigh, these varying views on the importance of viability and other moral and clinical thresholds.

### The social and structural realities behind later abortion

Our findings suggest that hospital obstetricians were more engaged with the medical and procedural aspects of abortion provision than with the social and structural factors shaping non-medically indicated abortion trajectories. This may be due to their limited exposure to such cases, since these are typically managed by, and referred to, specialized abortion clinics. Notably, the obstetricians rarely addressed the underlying factors leading to later abortion presentations or the consequences of abortion denial. Extensive research highlights the diverse and complex reasons why women seek abortions at more advanced stages of pregnancy, including delayed recognition of pregnancy, practical barriers to accessing services, changes in health or relationship circumstances, legal restrictions, financial constraints, limited knowledge of abortion laws, and delays in obtaining pregnancy tests [39–46]. Several studies have also described the harmful effects on women after local denial of, and consequent travel for abortion [47–50]. These harms are considered particularly problematic as they disproportionally affect more vulnerable abortion-seekers [51–54]. Evidence from Flanders indicates that individuals seeking abortion services after the first trimester are often younger, less educated, unemployed, and from migrant backgrounds [51]. These vulnerabilities disproportionately expose them to the impacts of gestational time limits and the associated harms of abortion-related travel. Despite the limited attention to social and structural factors, obstetricians seemed open to providing abortion beyond 12 weeks in cases where abortion-seekers faced psychosocial or psychological hardships, or where a medical assessment was involved. This reflects a tendency to medicalize later abortion requests – a phenomenon consistent with the medical orientation of the obstetric profession. We suggest that greater awareness of, and engagement with, sociological evidence on the lived realities of abortion-seekers could further enrich obstetricians’ perspectives on elective abortion before and beyond the first trimester.

### The classification of abortion and abortion refusal

Finally, we found that obstetricians believed the current time limit for elective abortion − and any potential extension − would have little to no impact on their professional roles or hospital practices. This reflects both their current focus and preference for providing terminations only in cases of severe medical conditions. While some individual obstetricians expressed a willingness to assist in second-trimester elective abortion services, our main findings suggest that the current approach of refusal/referral of elective abortions by Flemish hospital obstetricians would largely persist.

Notwithstanding the obstetricians’ perceptions, it is crucial to note that a time limit extension would alter the legal classification of refusals to carry out elective abortions up to the new limit. Refusal to perform abortion after 12 weeks in the absence of serious medical indications aligns with the current law. However, if the abortion limit were extended, such refusal would constitute conscientious objection to an otherwise lawful request. Such a legal reclassification is likely to prompt discussions on the professional responsibilities of hospital obstetricians, not only when later abortion is requested due to an unwanted pregnancy, but also when it is sought for ‘milder’ conditions, fetal sex selection, or psychosocial indications. In this context, the obstetricians may have underestimated the extent to which an extended elective abortion limit could affect their own service provision, and overestimated the rigidity of the distinction between elective abortion and abortion on medical grounds. Clinical-ethical guidance and continued debate within the medical profession will be crucial to navigate some of the challenges and opportunities associated with a time limit extension, as identified in this study.

## Strengths and limitations of this study

This qualitative study offers a unique provider perspective on gestational age limits for elective abortion, highlighting both opportunities and challenges associated with extending these limits beyond the first trimester. The findings can inform policymakers in ways that prioritize patient safety, enhance organizational efficiency, and secure broader support from the obstetric profession. Furthermore, the study highlights potential implications for the obstetric profession and the practice of medically indicated abortion, which have been largely overlooked in the time limit debate.

Notwithstanding these strengths, this study inevitably presents certain limitations. This study is restricted to the views of obstetricians who primarily focus on providing abortion on medical grounds working in hospital settings and, hence, does not directly capture the perspectives of pregnant individuals, providers of ‘elective’ abortion operating in dedicated abortion clinics and family planning centers, civil society organizations, or other relevant stakeholders. As the hospital-based obstetricians in this study were only rarely involved in terminating pregnancies in the absence of a medical condition, their views on ‘elective’ abortion were likely shaped by a certain bias. We hypothesize that their general commitment to supporting healthy, wanted pregnancies to term − viewing termination as a last-resort option in cases of medical *force majeure* − influenced their perspectives. Additionally, for reasons of feasibility and due to language barriers, this study focused on Flemish obstetricians and did not include the perspectives from obstetricians in the Walloon region of Belgium. In Wallonia, hospital obstetricians are more frequently involved in ‘elective’ abortion provision. Future research should include these other stakeholders to determine how they position themselves on the question of elective abortion provision beyond the first trimester.

## Conclusion

In considering a possible extension of Belgium’s 12-week elective abortion limit, obstetricians in Flanders primarily highlighted medical, moral, and logistical concerns. They less often addressed the social and structural factors influencing later abortion trajectories and impacting patient experiences. Our qualitative findings, supported by the literature, point to the need for implementation safeguards should a time limit extension be adopted, including the training of staff, appropriate and centralized facilities, specialized instruments, and sufficient caseloads to maintain expertise. We argue that greater attention to the lived experiences of abortion-seekers could broaden obstetricians’ perspectives and potentially increase their willingness to provide care before or after the current limit. Values clarification workshops, identified in the literature, emerge as a promising tool to foster empathetic care and to identify providers interested in pursuing specialized training in second-trimester abortion. Finally, our study emphasizes that refusing abortion within the extended period would constitute conscientious objection to a lawful intervention. An extension should therefore prompt the medical community to engage in clinical-ethical dialogue and develop guidance and policies for handling requests beyond strict medical grounds.

## Supporting information

S1 File The semi-structured topic guide used during interviews was added as a supplementary file. (PDF)
