# Supplementary material for: Obstetricians’ views on extending the 12-week abortion limit in Belgium: A qualitative study
Source: PLoS One. 2025 Jun 18;20(6):e0325434. doi: 10.1371/journal.pone.0325434 (PMC12176136; doi:10.1371/journal.pone.0325434)
Supplement: S1 File — (PDF) [file pone.0325434.s001.pdf]

Semi-structured topic guide – Obstetricians' decision-making processes, experiences, and views regarding abortion (=termination of pregnancy) after the first trimester[1]

Note that the interview topic guide covers subjects not reported in the paper submitted to PLOS ONE. Our paper discusses the answers to question 2.B, as well as those answers to question 1 and 2.A that were considered of relevance to question 2.B.

<table>
<tr><th>Guiding questions</th><th>Additional questions/prompts</th></tr>
<tr><td colspan="2">Introduction</td></tr>
<tr><td>- Ask full name of the participant
- Thank participant for their participation
- Introduce interviewer
- Explain the purpose of the research and the interview
- Emphasize confidentiality
- Indicate that participants can interrupt the conversation at any time if desired
- Explanation of informed consent
- Confirmation of signature informed consent
- Fill in list of demographic questions</td><td></td></tr>
<tr><td colspan="2">Introductory question</td></tr>
<tr><td>- We want to discuss termination of pregnancy. Is this something you regularly deal with in your department?</td><td></td></tr>
<tr><td colspan="2">Transition question</td></tr>
<tr><td colspan="2">- What factors influence and complicate the appropriateness of requests for termination of pregnancy after 14[1] weeks?</td></tr>
<tr><td>Core questions</td><td>Prompts</td></tr>
<tr><td>1.A What factors do you consider when deciding whether fetal conditions warrant or necessitate pregnancy termination after 14 weeks?

1.B What factors do you consider when deciding whether certain conditions in the pregnant woman warrant or necessitate pregnancy termination after 14 weeks?

[Question 1 is not the focus of this paper, but some answers to question 1 have been discussed in it due to their relevance to question 2.B]</td><td>1. To what extent do you consider the following factors in your deliberation of requests:
a. Clinical factors: severity, certainty, nature of conditions, fetus survival chances, prognosis, ... Discussion of the above elements for certain conditions?
b. Ethical factors: personal ethical views of the physician, religious beliefs, viability, ...
c. Legal factors: reference to current legal framework, proposed legislative changes, legal doctrine or jurisprudence, interpretation of legal conditions, reference to legal term, ...
d. Social factors: vulnerable background of pregnant woman, future child's upbringing environment, societal context for disability, ...
e. Timing-related factors: timing in pregnancy at the time of termination, timing of discovery of conditions, urgency in therapeutic termination, ...
f. Institutional factors: hospital policy, collaboration among colleagues, ...
g. Experience-related factors: physician's expertise with a certain termination method, outcome of previous decisions regarding continuation/termination of pregnancy, personal experience (e.g., knowing a child with a condition), ...</td></tr>
</table>

[1] Equals 12 weeks dated from conception.

2.A The law imposes certain procedural requirements for terminations after 14 weeks pregnancy. How do you experience and view these requirements in practice?<br><br>a. Six-day waiting period + starting point of the waiting period<br>b. Obligation to involve a second physician (institutionally anchored decision-making process?)<br>c. Conscientious objection + referral obligation<br>d. Registration obligation to the National Evaluation Committee for Pregnancy Termination<br>e. Information obligations, including information about adoption and contraception<br><br>*[Question 2.A is not the focus of this paper, but some answers to question 1 have been discussed in it due to their relevance to question 2.B]*<br><br>2.B The law imposes a 14-week limit for abortion on request.<br><br>a. How do you experience and view this temporal limit in practice?<br>b. What do you think of the political proposal for an extension of this limit?<br><br>*[Question 2.B is the focus of this paper]*	
**Closing question**	
- Do you think the current legal framework adequately meets your needs as an obstetrician?	
**Final comments from the participant**	
- Are there any other issues that haven't been addressed that you would like to discuss?	
